# The protective effects of flavonoids and carotenoids against diabetic complications—A review of *in vivo* evidence

**DOI:** 10.3389/fnut.2023.1020950

**Published:** 2023-03-24

**Authors:** Yannan Jin, Randolph Arroo

**Affiliations:** ^1^Leicester School of Allied Health Sciences, Faculty of Health & Life Sciences, De Montfort University, Leicester, United Kingdom; ^2^Leicester School of Pharmacy, Faculty of Health & Life Sciences, De Montfort University, Leicester, United Kingdom

**Keywords:** flavonoids, carotenoids, diabetic complications, inflammation, oxidative stress

## Abstract

Diabetes mellitus is a chronic metabolic disorder caused either by inadequate insulin secretion, impaired insulin function, or both. Uncontrolled diabetes is characterized by hyperglycemia which over time leads to fatal damage to both macro-and microvascular systems, causing complications such as cardiovascular diseases, retinopathy and nephropathy. Diabetes management is conventionally delivered through modifications of diet and lifestyle and pharmacological treatment, using antidiabetic drugs, and ultimately insulin injections. However, the side effects and financial cost of medications often reduce patient compliance to treatment, negatively affecting their health outcomes. Natural phytochemicals from edible plants such as fruits and vegetables (F&V) and medicinal herbs have drawn a growing interest as potential therapeutic agents for treating diabetes and preventing the onset and progression of diabetic complications. Flavonoids, the most abundant polyphenols in the human diet, have shown antidiabetic effects in numerous *in vitro* and preclinical studies. The underlying mechanisms have been linked to their antioxidant, anti-inflammatory and immunomodulatory activities. Carotenoids, another major group of dietary phytochemicals, have also shown antidiabetic potential in recent *in vitro* and *in vivo* experimental models, possibly through a mechanism of action similar to that of flavonoids. However, scientific evidence on the efficacy of these phytochemicals in treating diabetes or preventing the onset and progression of its complications in clinical settings is scarce, which delays the translation of animal study evidence to human applications and also limits the knowledge on their modes of actions in diabetes management. This review is aimed to highlight the potential roles of flavonoids and carotenoids in preventing or ameliorating diabetes-related complications based on *in vivo* study evidence, i.e., an array of preclinical animal studies and human intervention trials. The current general consensus of the underlying mechanisms of action exerted by both groups of phytochemicals is that their anti-inflammatory action is key. However, other potential mechanisms of action are considered. In total, 50 *in vivo* studies were selected for a review after a comprehensive database search *via* PubMed and ScienceDirect from January 2002 to August 2022. The key words used for analysis are type-2 diabetes (T2DM), diabetic complications, flavonoids, carotenoids, antioxidant, anti-inflammatory, mechanisms of prevention and amelioration, animal studies and human interventions.

## 1. Introduction

Diabetes is a chronic endocrine disease caused by either insulin deficiency, ineffective use of insulin or both (1). Diabetes is characterized by prolonged high blood glucose levels (hyperglycemia), which, if left uncontrolled, can result in serious damage to different tissues and organs, e.g., the eyes, kidneys and heart. Diabetes is conventionally classified into two types, i.e., type-I (insulin-dependent) diabetes and type-II (non-insulin dependent) diabetes (1). The current global prevalence of diabetes is estimated to be 9.3% (463 million people). It is projected to increase by 25% in 2030 and 51% in 2045, where regions with economies moving from low-to middle-income status will likely be mostly affected (2).

Type-II diabetes mellitus (T2DM) is the prevalent form of diabetes (around 90%) worldwide (3). Its pathogenesis features a lowered sensitivity of the insulin receptors on cells in the liver, skeletal muscles and adipose tissues, i.e., insulin resistance, resulting in a reduced uptake of glucose into its recipient tissues. This often triggers an initial increased insulin secretion (hyperinsulinemia) from beta-cells in pancreas, which however leads to beta-cell dysfunction at advanced stages with reduced insulin secretion (hypoinsulinemia), ultimately resulting in high blood sugar levels (hyperglycemia) (1). Type-2 diabetic patients are often found obese—80% prevalence rate. The treatment target for T2DM is to re-establish normal blood glucose and lipid levels (4).

Ineffective management of diabetes can lead to many complications, commonly cardiovascular diseases, kidney disease, nerve and eye damage, skin problems, increased susceptibility to infections and dental problems (5). Metabolic inflammation mediated by various pro-inflammatory cytokines (e.g., tumor necrosis factor-α-TNF-α) and chemokines (e.g., chemokine C-C motif ligand-2 and-5) has been recognized to be the leading cause of the onset and advancement of diabetic complications (6). At the initial stage of insulin resistance, macrophage-derived interleukin (IL)-1β can stimulate insulin secretion *via* promoting β-cell proliferation. However, as insulin resistance persists, the prolonged action of IL-1β stimulates the generation of a wide array of cytokines and chemokines such as IL-6, IL-8 and IL-33. These attract macrophages and immune cells to the pancreatic islet where β-cells are located, further triggering the IL-1β auto-stimulation cycle. The consequence of excessive production of cytotoxic factors from macrophages and IL-1β is the mass reduction of β-cell and its impaired function (7). Moreover, hyperglycemia casts additional inflammatory burden to the pancreatic islet by stimulating the production of pro-inflammatory cytokines that are regulated by the transcription factor, nuclear factor-kappa-B (NF-κB), the central regulator of inflammation. The systemic low-grade inflammation can markedly accelerate tissue injury from pancreatic islet to a wider vascular damage that mediates the progression of diabetic microvascular complications such as diabetic kidney disease, diabetic retinopathy and diabetic neuropathy (8, 9). This highlights the importance of suppressing the diabetes-induced immune-inflammatory responses in treating diabetes and preventing its progression to microvascular complications.

### 1.1. Diabetic complications

Diabetic complications are classified into macrovascular and microvascular complications (10). The former type comprises coronary artery disease, peripheral arterial disease and stroke; whist the latter is manifested into diabetic eye deterioration (retinopathy), kidney disease (nephropathy) and peripheral nerve damage (neuropathy) caused by changes in the thickness of the capillary basement membrane (11).

#### 1.1.1. Diabetic cardiovascular complications

Diabetes is recognized as an independent risk factor for cardiovascular diseases (CVD) including atherosclerotic coronary heart disease, ischemic heart disease, myocardial infarction and stroke (5). The pathogenesis of CVD involves the development of vascular endothelial dysfunction, atherosclerosis, hypertension, increased oxidative stress and chronic low-grade inflammation. The chronic inflammation and increased oxidative stress resulting from insulin resistance and hyperglycemia in diabetes create an atherothrombotic environment that promotes the development of endothelial dysfunction and atherosclerosis, eventually leading to diabetic cardiovascular complications (DCC). Other factors related to insulin resistance, such as dyslipidemia and hypertension, further elevate the risk of CVD in diabetes (12).

#### 1.1.2. Diabetic nephropathy

Diabetic nephropathy (DN) is considered one of the most common diabetic microvascular complications, prevailing between 30 and 50% of patients with diabetes. It is largely responsible for the end-stage renal failure in patients with diabetes (13). The clinical symptoms feature progressive proteinuria and a noticeable decline in glomerular filtration rate, indicative of deteriorating function of renal nephrons. Pathogenesis of DN involves the elevated glomerular basement membrane thickness, microaneurysm formation, mesangial nodule formation (Kimmelsteil–Wilson bodies), and other changes. Chronic inflammation is identified as the key causative factor for the DN and the advancement of the condition is closely related to a bundle of pro-inflammatory cytokines, such as IL-1, IL-6 and TNF-α (14). The recent research provides new insights in the therapeutic treatment for DN by targeting at inflammatory response and pro-inflammatory cytokines for DN.

#### 1.1.3. Diabetic retinopathy

Diabetic retinopathy (DR) is the primary cause of blindness in patients with diabetes. The DR occurs in around 35% (92.6 million) among diabetic adult patients, of which 28.4 million suffering from vision impairment (15, 16). It involves changes in retinal ganglion cells (RGCs). Patients with prolonged duration of diabetes and poor control of hyperglycemia and hypertension are prone to developing DR. The sustained diabetes-evoked inflammatory responses mediated by pro-inflammatory modulators (IL-1β, TNF-α) majorly contribute to the development of DR, consequently altering the retinal structure and impairing its function. In addition, growth factors including vascular endothelial growth factor (VEGF) and transforming growth factor beta (TGF-*β*), also play important roles in the development of DR. In general, the mechanistic basis of the development of DR resembles that for DN (17).

#### 1.1.4. Diabetic peripheral neuropathy

Diabetic peripheral neuropathy (DPN), as the most common long-standing diabetic neuropathy, affects the sensory and autonomic nervous system. More than half of the diabetic patients suffers from this complication (18). Symptoms of DPN are often manifested as pain and abnormal heat/cold sensitivity, making patients susceptible to increased risk of burns, injuries, foot ulceration, and the need for amputations (19). The pathogenesis of DPN is complex, primarily involving axonal atrophy, demyelination, blunted regeneration, and loss of neuronal fibers. Hyperglycemia and dyslipidemia in the presence of insulin resistance are the major contributors to DPN’s progression. The injury to peripheral nerves triggers enhanced inflammatory responses *via* the action of inflammatory cytokines such as TNF-α (20).

In general, the clinically diverse microvascular complications of diabetes share a similar root of their pathogenesis, i.e., all involving hyperglycemia and increased oxidative stress, inflammation, and formation of advanced glycation end products (AGEs).

### 1.2. Diabetes management

The goal of diabetes management is to achieve optimal glycemic control and prevent or delay the onset and progression of diabetic complications. Dietary and lifestyle modification are recommended approaches for managing diabetes, together with pharmacological interventions including oral hypoglycemic medications (e.g., glipizide, metformin, or acarbose) or ultimately insulin injections (21). Yet, diabetic patients commonly experience issues of taking anti-diabetic medications, such as hypoglycemia, weight gain, headache and nausea and the financial cost (21). Emerging preclinical and clinical evidence has demonstrated notable benefits of inflammation-targeted dietary therapies using natural products in treating hyperglycemia, β-cell dysfunction, and insulin resistance in diabetes (6, 10). Moreover, cohort studies have shown that greater intake of green leafy vegetables was associated with a reduction in risk of T2DM (22, 23). Notably, flavonoids and carotenoids, natural phytochemicals commonly found in edible fruits and vegetables (F&V), have been shown to mediate immune-inflammatory responses and modulate non-cytokine mediators such as reactive oxygen species and nitric oxide (NO), thus potentially protecting against diabetes-related complications (24–27). A large body of research has focused on the preventive effects of flavonoids and carotenoids against the immediate effects of T2DM (28–32), but their protective effects against diabetic complications have remained less studied, with clinical evidence emerging but still limited (33, 34). This review will primarily discuss the therapeutic potential of dietary flavonoids and carotenoids in preventing/treating diabetic complications based on *in vivo* evidence and examine the possible mechanistic bases involved with a particular focus on the immune-inflammation modulatory effects of those chemicals.

## 2. Flavonoids and flavonolignans

**Figure 1:**
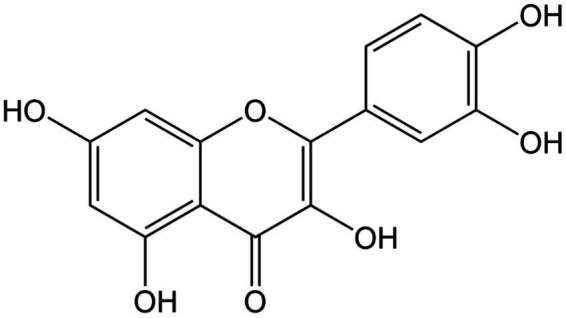
Chemical structure of quercetin.

**Figure 2:**
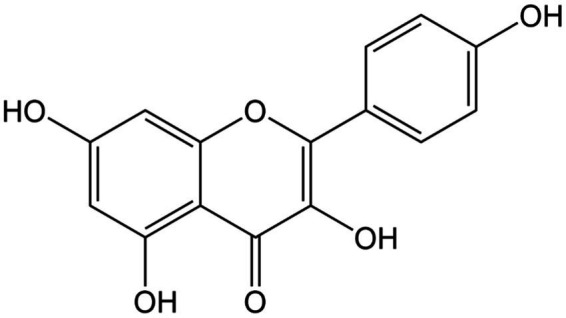
Chemical structure of kaempferol.

**Figure 3:**
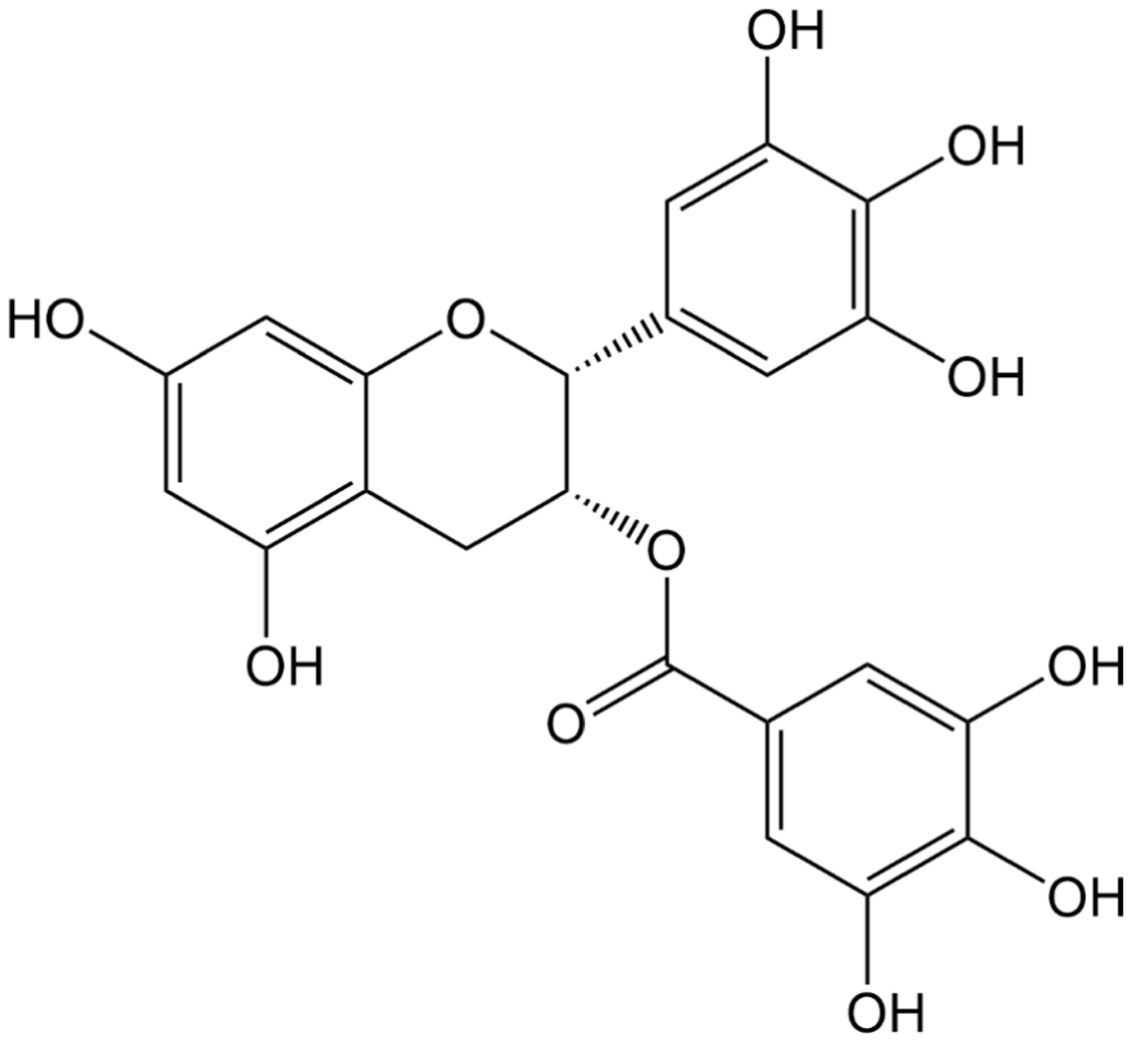
Chemical structure of epigallocatechin gallate.

**Figure 4:**
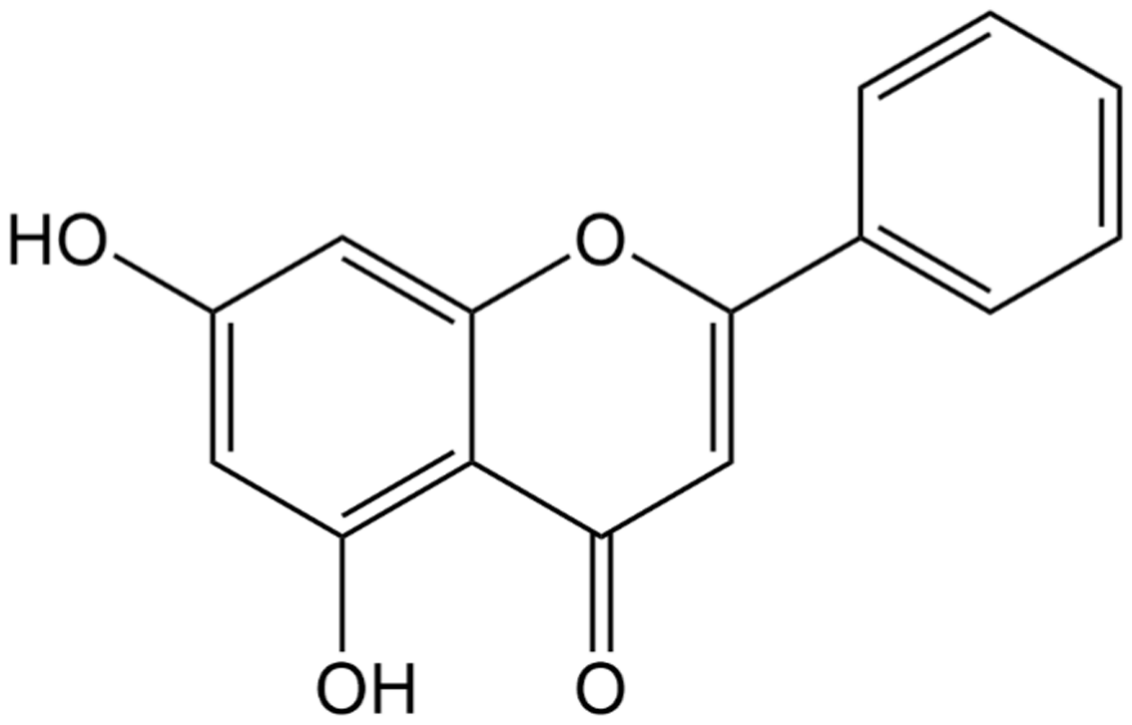
Chemical structure of chrysin.

**Figure 5:**
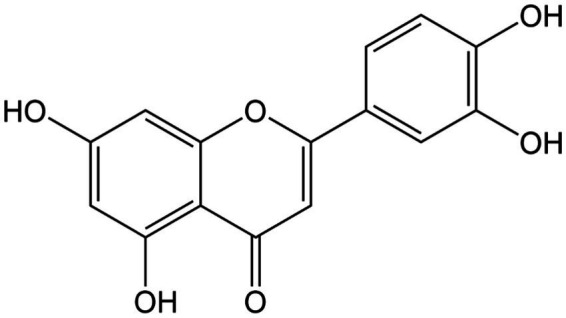
Chemical structure of luteolin.

**Figure 6:**
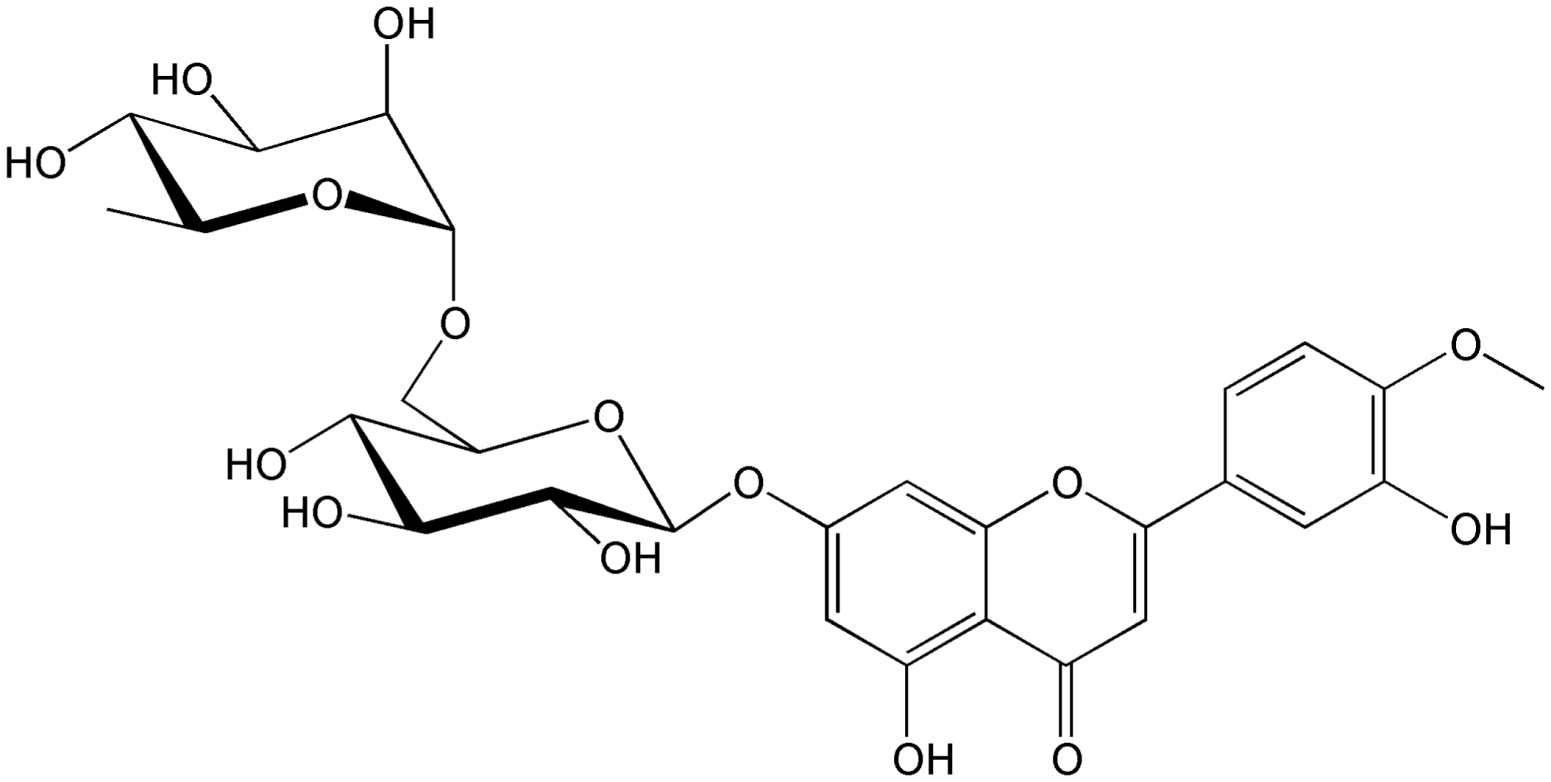
Chemical structure of diosmin.

**Figure 7:**
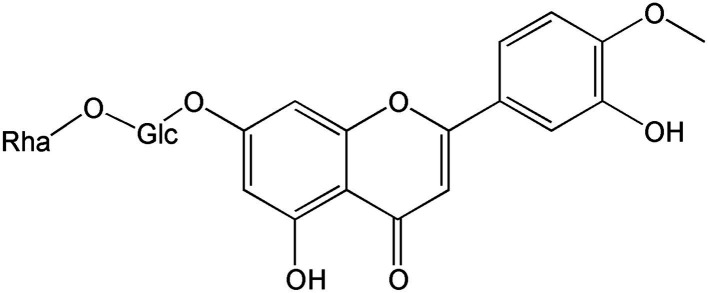
Chemical structure of diosmin-2.

**Figure 8:**
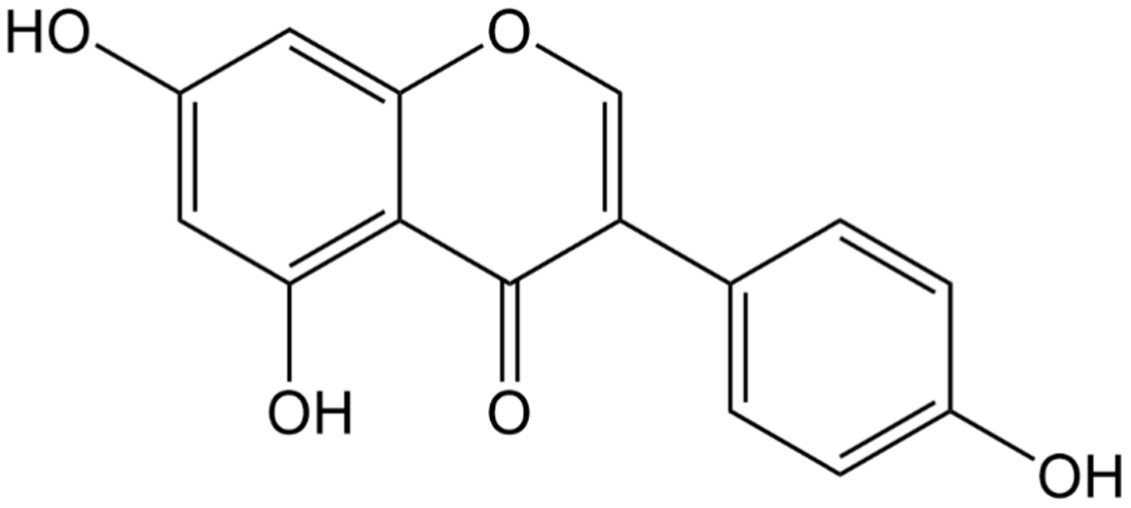
Chemical structure of genistein.

**Figure 9:**
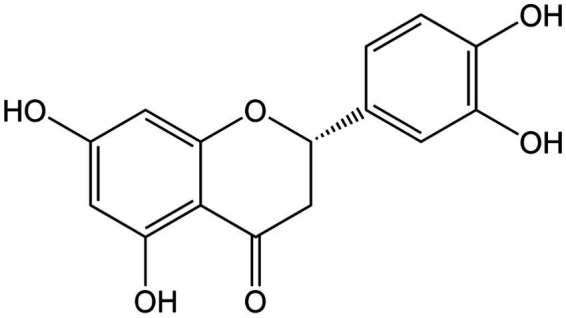
Chemical structure of eriodictyol.

**Figure 10:**
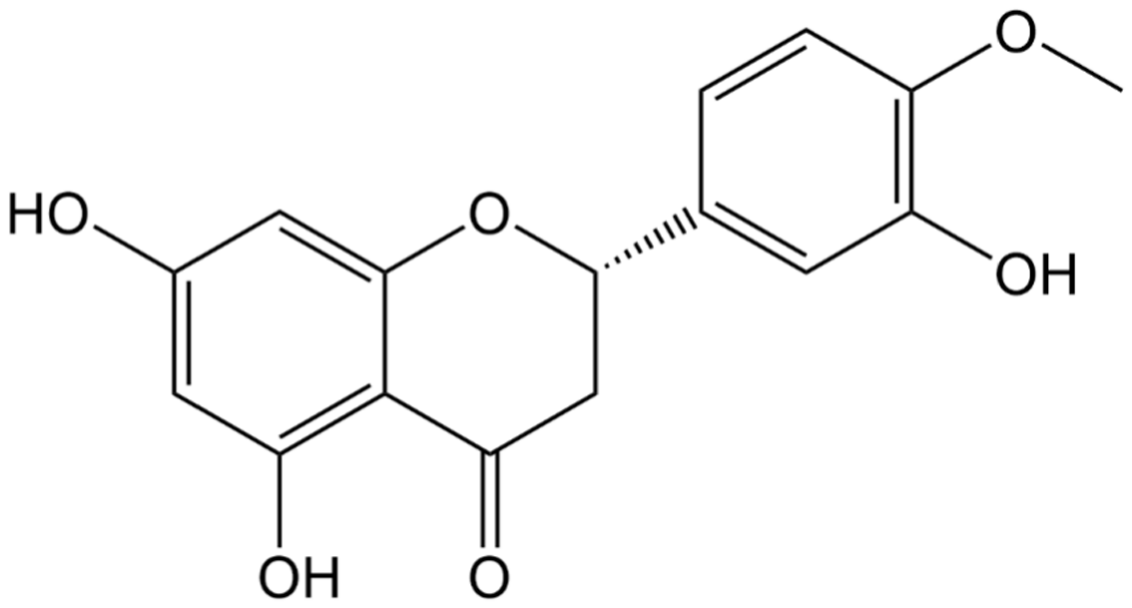
Chemical structure of hesperetin.

**Figure 11:**
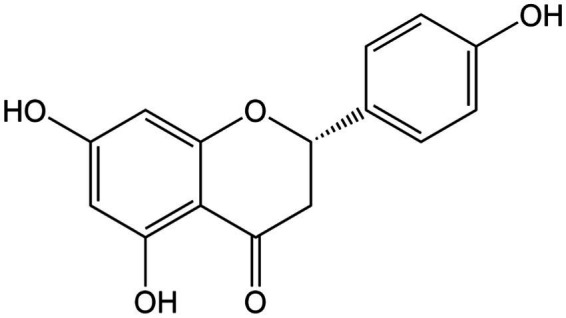
Chemical structure of naringenin.

**Figure 12:**
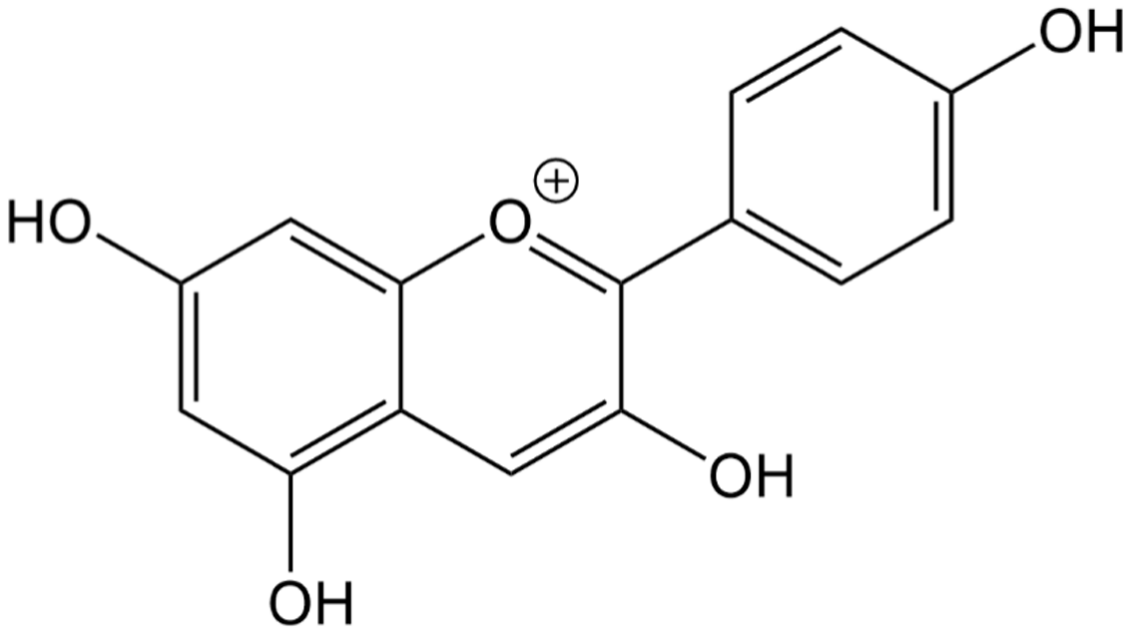
Chemical structure of anthocyanins (pelargonidin).

**Figure 13:**
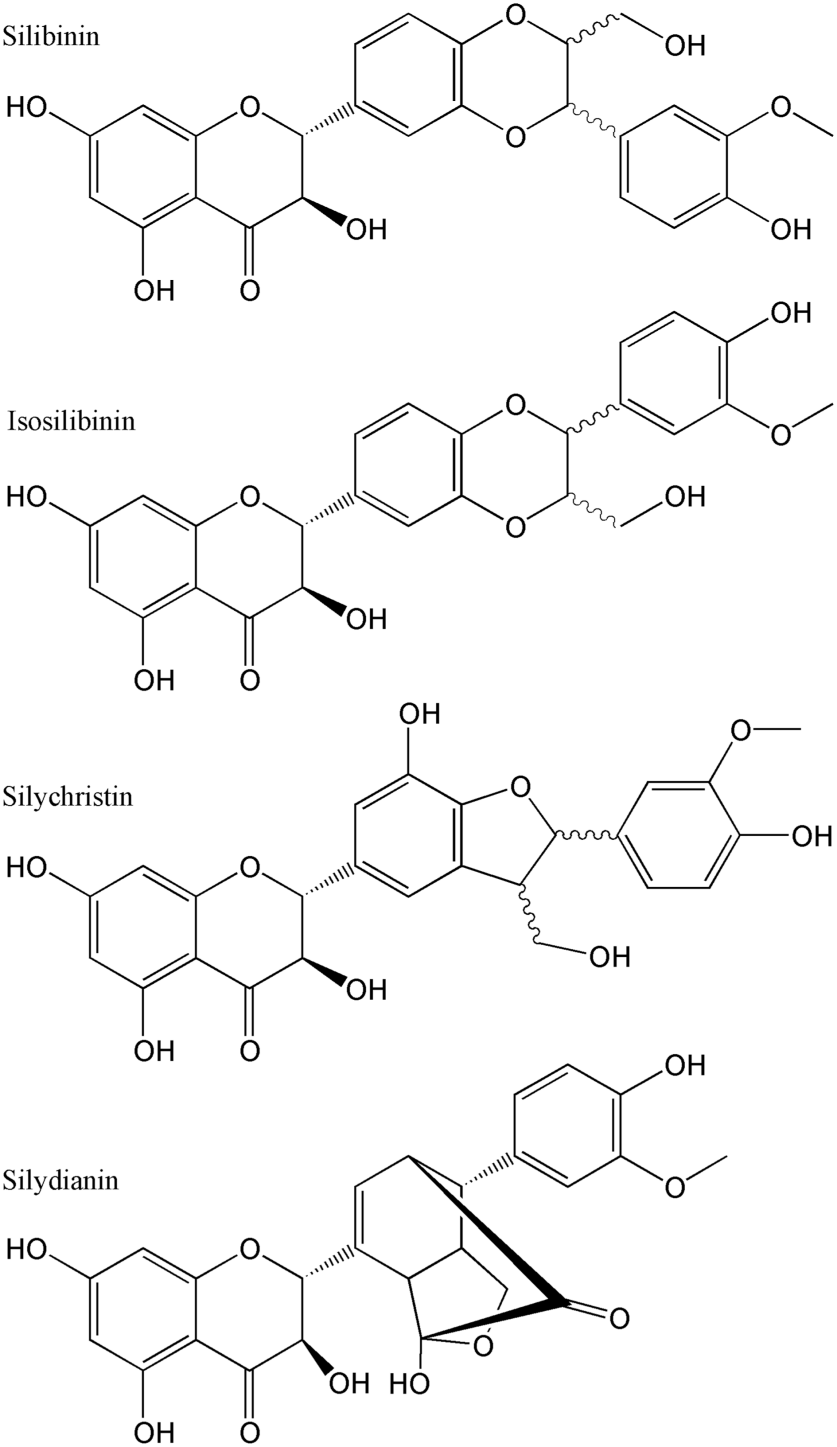
Chemical structures of some of the silymarin components.

Flavonoids are widely found as secondary metabolites in F&V, notably berries and citrus fruits, leafy vegetables, onions and beverages like tea and wine (35). Their polyphenolic structure features two benzene rings attached by a short three carbon chain. Flavonoids can be divided into sub-categories including flavonols (e.g., quercetin, kaempferol; Figures 1, 2), flavanols (e.g., epicatechin; Figure 3), flavones (e.g., chrysin, luteolin, and diosmin; Figures 4–7), isoflavones (e.g., genistein; Figure 8), flavanones (e.g., eriodictyol, hesperetin, and naringenin; Figures 9–11), anthocyanins (e.g., pelargonidin; Figure 12) (35). Silymarin is a standardized mixture of different flavonolignans extracted from the seeds of the milk thistle (*Silybum marianum* (L.) Gaertn.). It comprises a mixture of flavonolignans such as isosilibinin, silychristin, and silydianin (36) (Figure 13). The antidiabetic properties of certain flavonoids and flavonoligans have been widely reported, including hypoglycemic, antioxidant and anti-inflammatory effects and their modulatory role in cell signaling and immune-inflammation responses (37). The names and dietary sources of a few flavonoids that have shown antidiabetic functions in studies are presented in Table 1.

**Table 1:** Major sources of flavonoids and carotenoids used in the treatment of diabetes and its complications.

Compound group	Name of compound	Main natural sources	References
Flavonol	Quercetin	Apples, berries, Brassica vegetables, capers, grapes, onions, shallots, tea, and tomatoes, as well as many seeds, nuts, flowers, barks, and leaves	(38)
Flavonol	Kaempferol	Green leafy vegetables, including spinach and kale, and herbs such as dill, chives, and tarragon	(39)
Flavanone	Eriodictyol	Citrus fruits such as lemon, vegetables, and most of the medicinal plants	(40)
Flavone	Luteolin	Celery, parsley, broccoli, onion leaves, carrots, peppers, cabbages, apple skins, and chrysanthemum flowers	(41)
Flavone	Chrysin	Honey, propolis, and various medicinal plants and fruits such as bitter melon (Momordica charantia) or the wild Himalayan pear (Pyrus pashia)	(42)
Flavone	Diosmin	Citric fruits such as lemon	(43)
Isflavones	Genistein	Soy-based foods, such as soy cheese, soy milk or soy-based beverages, legumes such as broad beans and chick peas and certain fruits and nuts such as native cherry cultivars of Hungarian origin and pistachio nuts	(44)
Flavanol	Epigallocatechin gallate	Cranberries, strawberries, blackberries, kiwis, cherries, pears, peaches, apples, avocados, pecans, pistachios, and hazelnuts	(45)
Flavanone	Hesperetin	Citric fruits such as oranges, mandarins and tangerines	(46)
Flavanone	Naringenin	Grapefruit, tomatoes and tomato-based products	(46)
Flavonoid	Anthocyanins	Berries, currants, grapes, and some tropical fruits have high anthocyanins content. Red to purplish blue-colored leafy vegetables, grains, roots, and tubers	(47)
Flavonolignans	Silymarin	Milk thistle (*Silybum marianum*) seeds	(48)
Xanthophyll	Lutein	Green leafy vegetables (e.g., kale, spinach, broccoli, peas, and lettuce), zucchini and different kinds of squash, kiwi fruit, grapes, orange juice, egg yolks, maize, einkorn, Khorasan and durum wheat and corn	(49, 50)
Xanthophyll	Zeaxanthin	Green leafy vegetables (e.g., kale, spinach, broccoli, peas and lettuce), orange pepper, zucchini and different kinds of squash, kiwi fruit, grapes, orange juice, egg yolks, maize, einkorn, Khorasan and durum wheat and corn	(49, 50)
Xanthophyll	Astazanthin	algae, yeast, salmon, trout, krill, shrimp and crayfish	(51)
Carotene	Lycopene	Tomatoes, pink guavas, apricots, watermelons, and pink grapefruits	(52)
Carotene	β-carotene	Carrot, tomatoes, spinach, potatoes, sweet potatoes, red peppers, watermelon	(53)

## 3. Carotenoids

**Figure 14:**
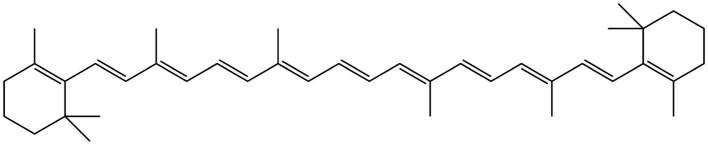
Chemical structure of β-carotene.

**Figure 15:**
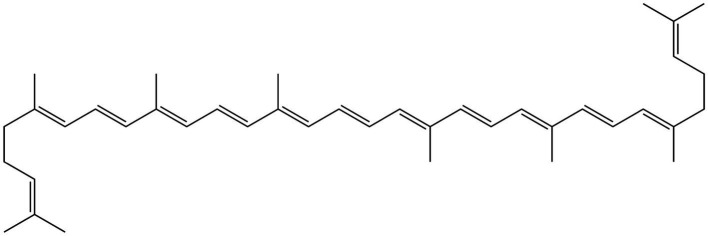
Chemical structure of lycopene.

**Figure 16:**
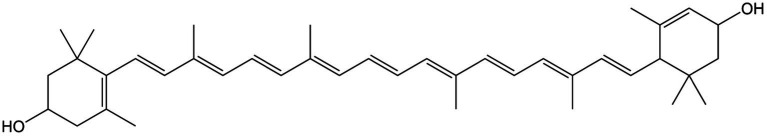
Chemical structure of lutein.

**Figure 17:**
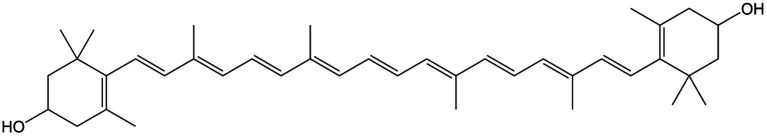
Chemical structure of zeaxanthin.

**Figure 18:**
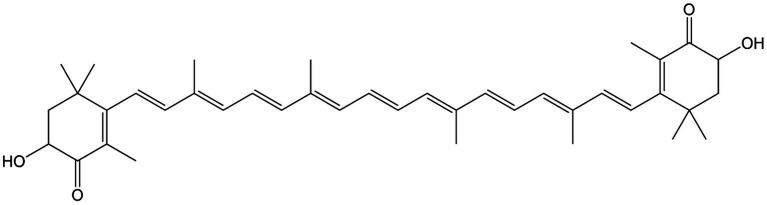
Chemical structure of astaxanthin.

Carotenoids are a diverse group of lipid-soluble pigments occurring in a wide array of photosynthetic organisms including plants, algae and cyanobacteria. To date, more than 650 types of carotenoids have been identified which all share the basic chemical structure of eight isoprene units, forming a C40 backbone (54). Carotenoids are subdivided into two types: carotenes, made only of hydrocarbons (e.g., carotenes and lycopene; Figures 14, 15), and xanthophylls (e.g., lutein, zeaxanthin and astaxanthin) which contain one or more oxygen atoms (Figures 16–18). Among the 30–40 of carotenoid metabolites present in human blood, β-carotene, lycopene, lutein, β-cryptoxanthin and zeaxanthin share the bulk (55). The chemical properties of carotenoids, notably their potent antioxidant capacity, have been well studied. Recently, there has been a growing interest in their biological activities in relation to their potential role in prevention of certain chronic diseases such as diabetes and related complications (56–58). Table 1 presents the names and natural sources of a few types of carotenoids with reported antidiabetic potential in animal and in clinical studies.

## 4. Evidence on protective effects of flavonoids against diabetic complications

### 4.1. Animal study evidence

The cardioprotective effects of flavonoids have been shown in diabetic animal models using a range of supplementation doses from 2 to 300 mg/kg body weight (bwt) /day with various durations (4–24 weeks; Table 2). Quercetin ameliorated diabetes-induced vasoconstriction and reduced the elevated blood pressure in diabetic rats when dosed at 50 mg/kg bwt daily for 6 weeks (59). The flavonol also inhibited diabetes-induced adventitial leukocyte infiltration, endothelial pyknosis and increased collagen deposition. Immunohistochemical assays indicated that the cardioprotective effects of quercetin could be attributed to its anti-inflammatory effects exhibited *via* its inhibition of aortic NF-κB and reducing the serum level of both TNF-α and C-reactive protein (CRP). It presented the potential to prevent the onset of DCC through interference with inflammatory pathways especially NF-κB signaling (59). Another flavonol, kaempferol, presented a dose–response effect at an ingestion level of 50 or 150 mg/kg/day for 10 weeks in attenuating hyperglycemia and hyperinsulinemia in streptozotocin (STZ)-induced diabetic rats. It showed potent anti-inflammatory effects by reducing nucleic and cystosolic levels of NF-κB, TNF-α and IL-6. It also altered the insulin signaling defect by inhibiting the phosphorylation of insulin receptor substrate-1 (IRS-1), IκB kinase α (IKKα) and IκB kinase β (IKKβ) (60). Oral ingestion of the isoflavone genistein in STZ-induced diabetic rats, at a higher dosage (300 mg/kg/day) and for a longer period (24 weeks), restored the integrity of their myocardia, accompanied by reducing diabetes-induced cardiac inflammation and oxidative stress *via* the reduction of TNF-α, CRP and TGF-*β1*. Furthermore, the results showed a notable amelioration of the ultrastructural degenerative changes in the diabetes-injured cardiac tissues (61). Supplementation with the flavone luteolin provided a similar cardioprotective effect in STZ-induced diabetic mice by preventing cardiac fibrosis, hypertrophy and dysfunction. The heart tissue injury was prevented by luteolin’s modulations of nuclear factor-erythroid two related factor two (Nrf2)-mediated-oxidative stress and NF-κB-mediated inflammatory responses (62). STZ-induced diabetic rats treated with epigallocatechin-3-gallate (ECGC) exhibited a decreased level of Deoxyribonucleic acid (DNA) damage in the myocardium. Histological examinations further indicated the cardioprotective effect of EGCG through preventing diabetes-induced damage, i.e., reducing the cardiac fibrosis in cardiac tissues whilst improving most of the cardiac muscle fibers. Other diabetic phenotypes including hyperglycemia, dyslipidemia and increased oxidative stress were all significantly improved *via* ECGC’s antihyperglycemic, antidyslipidemic, and antioxidative actions (63). Lastly, STZ-induced diabetic rats, fed with anthocyanins (250 mg/kg/day) for 4 weeks, showed a significant restoration of their deteriorating cardiac functions with a remarkable reduction in their cardiac hypertrophy and fibrosis, compared to the controls (64).

**Table 2:** Protective effects of flavonoids against diabetic complications—animal study evidence.

Flavonoid name	Administration, Dosage form	Experimental design	Treatment	Duration	Upregulation	Downregulation	References
Quercetin	Oral, reconstituted solution	STZ-induced diabetic rats	50 mg/kg/day	6 weeks		1. Reduction of serum TNF-α and CRP levels 2. Reduction in the activation of the transcription factor NF-κB 3. Lowering SBP 4. Amelioration of the exaggerated contractile responses of aorta to PE and KCl 5. Prevention of leukocyte infiltration in the adventitia, inhibition of endothelial cells pyknosis and reduction in collagen deposition within aorta sections	(59)
Kaempferol	Oral, reconstituted solution	STZ-induced type 2 diabetic rats fed HFD	50, 150 mg/kg/day	10 weeks	Enhanced the insulin-induced glucose-lowering effect	1. Reduction in TC, TG, LDL-C, and NEFA levels 2. Decreased serum TNF-α and IL-6 levels	(60)
Genistein	Oral, reconstituted solution	STZ-induced diabetic rats	300 mg/kg/day	24 weeks	Augmentation of total antioxidant reserve of the hearts.	1. Reduction in TNF-α, CRP, and TGF-β-1 2. Amelioration of the ultrastructural degenerative changes in the cardiac tissues	(61)
Luteolin	Oral, reconstituted solution	STZ-induced diabetic mice	20 mg/kg/day	15 weeks		Prevention of cardiac fibrosis, hypertrophy, and dysfunction in diabetic mice	(62)
ECGC	Oral, reconstituted solution	STZ-nicotinamide-induced diabetic rats	2 mg/kg/day on alternate days	1 month	1. Increased levels of SOD and CAT activities and GSH content in cardiac tissue 2. Significant improvement in most of the cardiac muscle fibres	1. Suppressed oxidative stress by reducing the levels of superoxide, 4-hydroxynonenal and protein carbonyl 2. Decreased level of DNA damage in the myocardium 3. Ameliorated the diabetes-induced damage in heart tissues 4. Reduction in the cardiac fibrosis in cardiac tissues 5. Decreased levels of IL-1β, IL-6 and TNF-α and adhesion molecules ICAM-1 and VCAM-1 6. Reduction in the number of apoptotic and necrotic cells	(63)
Anthocyanins	Oral, reconstituted solution	STZ-induced diabetic mice	250 mg/kg/day	4 weeks		Reduction in cardiac hypertrophy and fibrosis	(64)
Hesperetin	Oral, reconstituted solution	STZ-induced diabetic rats	100 mg/kg/day	24 weeks	Restored retinal GSH levels close to normal levels	1. Reduction of TNF-α and IL-1β in retinae 2. Inhibition of the expression of caspase-3, GFAP and AQP4 in retinae 3. Prevention of photoreceptor degeneration	(65)
Eriodictyol	Oral, reconstituted solution	STZ-induced diabetic rats	10 mg/kg/day	10 days		1. Reduction of retinal TNF-α, ICAM-1, VEGF and eNOS 2. Suppressed diabetes-related lipid peroxidation and the blood-retinal-barrier breakdown	(25)
Naringenin	Oral, reconstituted solution	Diabetic mice	1 and 2% of the diet	10 weeks		1. Reduction of IL-1β, IL-6, MCP-1, and TGF-β-1 2. Lowered protein kinase C activity and suppressed NF-κB p65 activity, mRNA expression, and protein production in kidney	(66)
Chrysin	Oral, reconstituted solution	HFD/STZ-induced type 2 diabetic rats	40 mg/kg/day	16 weeks	Improved renal pathology	1. Reduction of renal TNF-α expression and NF-κB activation 2. Suppressed TGF-β-1, fibronectin and collagen-IV protein expressions in renal tissues. 3. Reduction of serum IL-1β and IL-6	(67)

STZ, streptozotocin; TNF-α, tumor necrosis factor alpha; hs-CRP, high-sensitivity-CRP; NF-κB, Nuclear Factor kappa-light-chain-enhancer of activated B cells; SBP, systolic blood pressure; Phenylephrine, PE; HFD, high-fat-diet; TC, total cholesterol; TG, triglycerides; LDL-C, low-density-lipoprotein-C; NEFA, Non-Esterified Fatty Acids; IL-6, interleukin-6; TGF-β-1, ECGC, Epigallocatechin Gallate; transforming growth factor-β-1; SOD, superoxide dismutase; CAT, catalase; GSH, glutathione; IL-1β, interleukin-1β; ICAM-1, Intercellular Adhesion Molecule-1; VCAM-1, vascular cellular adhesion molecule-1; GFAP, glial fibrillary acidic protein; AQP4, aquaporin-4; VEGF, vascular endothelial growth factor; eNOS, endothelial nitric oxide synthase; MCP-1, monocyte chemoattractant protein-1.

The protective effects against DR were also seen with other types of flavonoids (Table 2). Hesperetin prevented the onset of DR in a STZ-induced diabetic rat model. The supplementation at 100 mg/kg/day for 24 weeks restored levels of the retinal antioxidant peptide glutathione (GSH) close to non-diabetic levels. Furthermore, hesperetin considerably reduced the retinal levels of inflammatory cytokines including TNF-α and IL-1β and hampered the expression of caspase-3, glial fibrillary acidic protein (GFAP) and aquaporin-4 (AQP4) in retinae which play a key role in retinal degeneration, and prevented the photoreceptor degeneration (65). Eriodictyol acted on a different mechanism in its protective role against DR in diabetic rats. The treatment attenuated the degree of retinal inflammation and plasma lipid peroxidation and prevented the breakdown of the Blood-Retinal-Barrier (BRB) in early STZ-induced diabetic rats. Notably, the reduction in retinal TNF-α, Intercellular Adhesion Molecule 1 (ICAM-1), vascular endothelial growth factor (VEGF), and endothelial nitric oxide synthase (eNOS) followed a dose–response manner using ingestion levels of 0.1, 1 and 10 mg/kg/day, with the last dosage being the most effective in DR prevention (25).

The anti-inflammatory activities of flavonoids have also been manifested in their protection against DN (Table 2). In diabetic mice, naringenin, when supplemented at 1 and 2% of their diet for 10 weeks, attenuated DN *via* its anti-inflammatory and antifibrotic activities. Both treatments significantly decreased the renal level of interleukin (IL)-1β, IL-6, and monocyte chemoattractant protein (MCP)-1, lowered protein kinase C (PKC) activity and suppressed the NF-κB p65 activity, mRNA expression, and protein production in kidney. The higher dose of 2% further decreased renal formation and expression of type IV collagen, fibronectin, and TGF-*β1*. Both doses increased the deposit of naringenin in the liver and kidney of diabetic mice (66). The findings suggested that naringenin may be helpful for preventing diabetic nephropathy. In a similar experimental setting, diabetic mice fed on high-fat-diet experienced improved renal pathology, abrogated renal dysfunction and oxidative stress upon an intervention with chrysin at 40 mg/kg/day for 16 weeks. Chrysin’s protective effect against DN is specifically mediated through its anti-inflammatory effects in the kidney targeting the TNF-α pathway (67).

### 4.2. Clinical evidence

Few human intervention studies thus far have shed light on the promising potential of flavonoids in tackling diabetic phenotypes and its complications. Flavonoids used in those studies was either dosed in their single or mixed isolation forms, or consumed as a whole food among diabetic patients with or without complications developed (Table 3). In a dietary intervention among 31 patients with T2DM, a 6-week daily intake of 500 mg of hesperetin significantly decreased the systolic blood pressure (SBP), diastolic blood pressure (DBP), levels of inflammatory markers including TNF-α, IL-6 and high sensitivity CRP (hs-CRP) and increased the serum total antioxidant capacity (TAC). There were no adverse events or side effect reported throughout the hesperidin supplementation. These results indicate hesperetin’s anti-inflammatory potential, and possible role in helping diabetic patients control their blood pressure naturally and safely (68). The mechanistic basis of the observed effects might have been further explained if the analyses of endogenous antioxidant enzymes such as superoxide dismutase (SOD) and GSH and serum NO level were included in the study design. ECGC supplementation at a dosage of 300 mg/day for 8 weeks remarkedly decreased the level of fasting blood glucose (FBG), hs-CRP as well as Body Mass Index (BMI) compared to baseline, among 50 patients with T2DM (69). The anti-inflammatory effect of ECGC was mediated through its inhibition of NF-κB pathway and suppression of the production of chemokines and inflammatory adipokines (74–76). The findings suggested a potential role of ECGC in improving glycemic control and inflammation status in T2DM. Unfortunately, glycosylated hemoglobin (HbA1c) levels were not considered in the analysis and only a relatively small cohort was recruited, i.e., 50 subjects in the intervention group. Care should be taken in order not to overinterpret the results of this study. The cardioprotective effect of flavonoids in diabetes was also observed when dosed as a whole food to male patients with T2DM. Upon daily intake of 22 g of freeze-dried blueberries (equivalent to one cup of fresh blueberries) for 8 weeks, in addition to habitual diet, there was a notable improvement in the cardiometabolic parameters of the 52 participants, shown as the reduced serum levels of HbA1c and fructosamine, total cholesterol (TC), low-density-lipoprotein-C and triglycerides. However, other parameters including FBG, insulin, CRP and high-density-lipoprotein cholesterol (HDL-C) and blood pressure were not affected by intervention (70). This study was one of the very first clinical studies examining the effect of freeze-dried blueberries in treating diabetic conditions. Again, sample size was a limiting factor of this trial that may have hindered the detection of differences in some key biomarkers between the intervention and placebo groups. Also, the fiber intake differed between the two groups, with the freeze-dried blueberry intervention containing 5.2 g of fiber and the placebo containing 0.3 g. Given an increased fiber intake can independently improve glycemic response (77, 78), further consideration is needed in the study design to identify the key influencer (i.e., either anthocyanins and phenolics from blueberries, or fiber content) on the glycemic control. Also, the overall efficacy observed could be better interpreted if data on the bioavailability of anthocyanins and other phenolics in blueberries were made available, as the presence of DN largely among participants at the start of the trial might have affected the bioavailability and functionality of the polyphenols consumed (79).

**Table 3:** Protective effects of flavonoids against diabetic complications—clinical evidence.

Flavonoid name	Administration, Dosage form	Experimental design	Treatment	Duration	Results		References
					Upregulation	Downregulation	
Hesperetin	Oral, dietary supplement	Double-blind RCT in 31 patients with T2DM	1 capsule daily (each contains 500 mg hesperetin)	6 weeks	Increased the serum TAC	Greater reduction in the percent change of SBP, DBP, mean arterial BP and inflammatory markers (TNF-α, IL-6, and hs-CRP) in Hesperidin group comparing to the control group	(68)
ECGC	Oral, dietary supplement	Double-blind, RCT in 50 patients with T2DM	Each capsule contains 150 mg, twice daily	8 weeks		1. Decreased levels of FBG 2. Reduced levels of hs-CRP	(69)
Anthocyanins	Oral, freeze-dried blueberries	Double-blind, parallel RCT in 52 patients with T2DM	22 g with 11 g taken with each of breakfast and evening meals along with typical diet	8 weeks		1. Reduction in the levels of HbA1c and fructosamine 2. Reduced levels of serum TC, LDL-C and triglycerides	(70)
Silymarin	Oral, tablets (each contain 140 mg of silymarin)	Double-blind, 2-arm parallel RCT in 30 patients with T2DM and diabetic nephropathy	3 × 140 mg silymarin	3 months		1. Higher decrement of UACR in the silymarin group than placebo group 2. Decreased urinary levels of TNF-α and urinary and serum levels of MDA	(71)
Flavones/flavonols, flavanones/dihydroflavonols, and phenolic compounds	Oral, Iranian propolis capsules (each contains 500 mg of propolis)	Double-blind RCT in 50 patients with T2DM	2 × 500 mg daily	90 days	Increased level of HDL-C	1. Reduction in HbA1C and insulin 2. Decreased levels of IL1-β, hs-CRP and TNF-α 3. Reduction in the blood urea nitrogen	(72)
Diosmin, *Centella asiatica* and Melilotus *officinalis*	Oral, dietary supplement	RCT with 35 patients with T2DM and diabetic CME	1 pill daily (containing 300 mg of diosmin, 15 mg of *Centella asiatica* and 160 mg of Melilotus *officinalis*)	36 months		A greater retinal sensitivity was present at month 12, 24, and 36 compared to the placebo group	(73)

RCT, randomized controlled trial; T2DM, type-2 diabetes mellitus; SBP, systolic blood pressure; DBP, diastolic blood pressure; BP, blood pressure; TNF-α, Tumor Necrosis Factor alpha; IL-6, interleukin-6; hs-CRP, high-sensitivity C-reactive protein P; ECGC, epigallocatechin-3-gallate; FBG, fasting blood glucose; HbA1C, glycosylated hemoglobin A1C; TC, total cholesterol; LDL-C, low-density lipoprotein cholesterol; UACR, Urinary albumin-creatinine ratio; MDA, malondialdehyde; HDL-C, high-density lipoprotein cholesterol; IL-1β, interleukin-1β; CME, cystoid macular edema.

Silymarin exhibited positive effect in preventing the progression of DN among 30 patients with T2DM and macroalbuminuria (urinary albumin excretion >300 mg/24 h) at a dosage level of 140 mg of silymarin taken thrice daily for 3 months. The intervention resulted in a higher decrement of urinary albumin-creatinine ratio (UACR) from baseline level compared to that of the placebo group, and also a significant reduction in the urinary levels of TNF-α and both urinary and plasma levels of malondialdehyde (MDA)—a marker of lipid peroxidation compared to placebo group (71). The results suggested silymarin be used as a novel agent to treat DN based on its antidiabetic and antioxidant potentials. Showing the similar renal-protective effects, the daily ingestion of a mixture of flavonoids (i.e., flavones/flavonols, flavanones/dihydroflavonols, and phenolic compounds) contained in Iranian propolis capsules at the level of 1,000 mg for 90 days majorly improved the diabetic phenotype, elevated the HDL-C and prevented renal dysfunction in 50 diabetic patients. The beneficial effects were observed through the reduction in postprandial blood glucose, serum insulin, insulin resistance, and inflammatory cytokines at the end of intervention (72). The effects of propolis may be ascribed to the synergistic actions of multiple compounds instead of isolated individual ones, however, the effective components and their doses, within the capsules, were not identified in the study (80). In another clinical trial using a mixture of flavonoids containing 300 mg of diosmin, 15 mg of *Centella asiatica* and 160 mg of Melilotus *officinalis*, the supplementation among 35 patients with T2DM and diabetic cystoid macular edema (CME) but without macular thickening remarkably restored the retinal sensitivity at months 12, 24, and 36 compared to the untreated group. But no differences in HbAc1, blood pressure, microalbuminuria, visual acuity, mean central retinal thickness, and stability of fixation were found between test and placebo groups during follow-up. No side effects from the intervention were reported, suggesting the safety and potential efficacy of using the compound mix as a natural agent for preventing DR (73).

## 5. Evidence on protective effects of carotenoids against diabetic complications

### 5.1. Animal study evidence

Lutein, zeaxanthin, lycopene and astaxanthin are the most-studied carotenoids in diabetic animal models for their putative therapeutic effects in preventing or treating diabetic complications, commonly DR (Table 4). In a mice model with an early stage of T2DM, the supplementation of 1% (kcal) wolfberry rich in lutein and zeaxanthin (a.k.a. Goji berry—*Lycium barbarum* L.) to the background diet for 8 weeks ameliorated mouse retinal abnormality *via* restoring the thickness of the entire retina, the structure of the inner nuclear layer and photoreceptor layer, and the integrity of retinal pigment epithelia (RPE), and the ganglion cell number in retina (81). The study further investigated the mechanism of wolfberry’s action on retinal structure restoration in diabetic mice by analyzing the retinal cell lysates of those mice treated with wolfberry supplements. The findings revealed that the wolfberry supplement downregulated the expression of endoplasmic reticulum (ER) stress biomarkers including binding immunoglobulin protein (BiP), protein kinase RNA-like ER kinase (PERK), activating transcription factor 6 (ATF6), and caspase-12. In contrast, the supplement restored the activities of AMP-activated protein kinase (AMPK) that regulates metabolic control in dedicated tissues such as the liver, muscle and fat (675). Similar effects were also exerted on, thioredoxin and manganese superoxide dismutase (MnSOD) as endogenous antioxidants (89, 90), and Forkhead O transcription factor 3α (FOXO3α). FOXO3 α protects cells from oxidative stress in diabetes upon activation (91, 92). ER stress majorly contributes to the cellular oxidative damage in the diabetic retina and eventually retinal cell apoptosis as a result of hyperglycemia and insulin resistance (93). Therefore, attenuating ER stress through modulating the expression of its sensor proteins/biomarkers, as the effect exerted by wolfberry supplement in this study, has been considered as one of the novel targets for diabetic therapy (94). Regarding the bioactive components in wolfberry that may have contributed to its retinal protective effect in the study, it was further confirmed through an additional *in vitro* experiment in the same study that the retinal-protective effect of wolfberry was at least partially due to its lutein and/or zeaxanthin content that showed to attenuate ER stress in ARPE-19 cells exposed to a high glucose challenge (81). In another study using diabetic mice, lutein on its own showed adjuvant effect in treating diabetes-induced oxidative stress in both retina and hippocampus (82). Hippocampus as part of the central nervous system is specifically susceptible to prolonged-hyperglycemia-induced alterations (95), i.e., apoptosis-induced neuronal loss leading to cognitive impairment after 8 months of diabetes, as reported in diabetic rat models (96). This study confirmed an initial impairment of hippocampus in untreated diabetic rats based on the positive correlation observed between the HbA1c values and hippocampal dysfunction. Also, retinal dysfunction was detected after 15 days of diabetes in rats. Lutein’s intragastrical ingestion at 0.2 mg/kg/day for 14 days restored the adverse changes in both retina and hippocampus of the diabetic rats. The protective effects were related to lutein’s antioxidant and anti-inflammatory activities, exhibited *via* its prevention of increases in oxidative stress (measured by the MDA and GSH concentrations and glutathione peroxidase (GPx) activity) and NF-κB activity in both diabetic retina and hippocampus (82). Zeaxanthin, on the other hand, also demonstrated the potential of preventing the development of retinopathy in diabetic rats when supplemented alone at 0.02% or 0.1% (w/w) for 8 weeks. It was found to share the similar mechanistic basis to that of lutein in its protective role (83).

**Table 4:** Protective effects of carotenoids against diabetic complications—animal study evidence.

Carotenoid name	Administration	Experimental design	Treatment	Duration	Results		References
					Upregulation	Downregulation	
Lutein and zeaxanthin	Oral, diet	Transgenic db/db type-2 diabetic mice	Formulated diet containing 1% (kCal) of wolfberry	8 weeks	1. Improved the thickness of the whole retina 2. Restored the inner nuclear layer and photoreceptor layer, the integrity of RPE, and the ganglion cell number		(81)
Lutein	Gastric tube	Alloxan-induced mice	0.2 mg/kg/day	14 days	Restored retinal and hippocampal levels of GSH and GPx activity compared to control group	1. Reduced serum, retinal and hippocampal levels of MDA 2. Prevention of increase in NFκB activity in the diabetic retina and hippocampus	(82)
Zeaxanthin	Oral, dietary supplement	STZ-induced diabetic rats	0.02% or 0.1% (w/w) zeaxanthin	8 weeks	1. Increased GSH/GPx activity 2. Elevated mitochondrial SOD	1. Inhibited diabetes-induced retinal oxidative damage 2. Prevented diabetes-induced elevation in retinal VEGF and ICAM-1 3. Decreased NFkB activity	(83)
Lycopene	Oral, solution	STZ-induced diabetic rats	20 mg/kg/day	8 weeks	1. Increased level of HDL-C 2. Increased Akt/PKB phosphorylation and SOD activity in diabetic renal tissues	1. Decreased levels of blood urea nitrogen, 24 h urea protein and creatinine. 2. Reduction in serum TC, TG, and LDL-C 3. Decreased levels of MDA content and expression of CTGF	(84)
Lycopene	Oral, solution	STZ-induced diabetic rats	5, 10, and 15 mg/kg/day	10 weeks	Increased otal antioxidative capacity and the activities of antioxidants including CAT, SOD, and GPx	1. Decreased FBG 2. Decreased oxidative stress biomarkers incl. GHb, ox-LDL, and MDA 3. Decreased TNF-α and CRP	(85)
Astaxanthin	Oral, dietary supplement	Diabetic db/db mice	0.02% (w/w) astaxanthin	12 weeks		1. Lowered blood glucose level 2. Inhibition of increases in urinary albumin and 8-OHdG 3. Reduced the mesangial area/total glomerular area ratio	(86)
Astaxanthin	Oral, dietary supplement	Diabetic db/db mice with diabetic retinopathy	25 or 50 mg/kg/day	8 weeks		1. Inhibited apoptosis of RGCs 2. Decreased levels of oxidative stress markers, including superoxide anion, MDA, 8-OHdG and MnSOD activity in the retinal tissue	(87)
Astaxanthin or lutein	Oral, solution	STZ-induced diabetic rats	0.6 mg/kg or 3 mg astaxanthin, or 0.5 mg/kg lutein daily	6 weeks	Increased levels of antioxidant enzymes (heme oxygenase-1 and peroxiredoxin)	1. Reduced levels of oxidative stress mediators (8-hydroxy-2′-deoxyguanosine, nitrotyrosine, and acrolein) in ocular tissues 2. Reduced levels of inflammatory mediators (ICAM-1, MCP-1, and fractalkine) 3. Reduced activity of the NF-κB	(88)

RPE, retinal pigment epithelia; GSH, glutathione; GPx, glutathione peroxidase; MDA, malondialdehyde; NF-κB, nuclear factor kappa-light-chain-enhancer of activated B cells; STZ, streptozotocin; HDL-C, high-density lipoprotein cholesterol; PKB, protein kinase B; SOD, superoxide dismutase; TC, total cholesterol; TG, triglycerides; LDL-C, low-density lipoprotein cholesterol; MDA, malondialdehyde; CTGF, connective tissue growth factor; CAT, catalase; FBG, fasting blood glucose; GHb, glycosylated hemoglobin; ox-LDL, oxidized-LDL; TNF-α, tumor necrosis factor alpha; CRP, C-reactive protein; 8-OHdG, 8-hydroxydeoxyguanosine; RGCs, retinal ganglion cells; MnSOD, manganese superoxide dismutase; ICAM-1, intercellular adhesion molecule-1; MCP-1, monocyte chemoattractant protein-1.

The efficacy of lycopene in preventing DN has been tested in several diabetic rat models, using a daily dose range between 5 and 20 mg/kg (Table 4). After an oral or intragastrical feeding for 8 or10 weeks in diabetic rats, lycopene prevented diabetes-induced renal lesions and improved renal function *via* attenuating oxidative stress by increasing the activities of endogenous antioxidants such as SOD and GPx and decreasing MDA and oxidized (ox)-LDL, and inhibiting NF-κB signal pathway. In addition, lycopene exhibited anti-hyperglycemia and anti-dyslipidemia properties by decreasing the FBG and modulating plasma levels of TC, LDL-C, HDL-C and triglycerides (84, 85). The findings support lycopene’s protective role against diabetic progression and further complications through various mechanisms.

Astaxanthin has demonstrated both protective potentials against DN and DR in diabetic db/db mice as a model of T2DM (Table 4). An oral dosage of 0.02% (w/w) astaxanthin for 12 weeks inhibited the progression and acceleration of diabetic nephropathy in diabetic db/db mice and also lowered blood glucose level. The antioxidative activity of astaxanthin attenuated the renal oxidative stress and prevented renal cell damage (86). The retinal protective effect of astaxanthin was further confirmed in another study using diabetic db/db mice with DR developed. The treatment of either 25 or 50 mg/kg/day for 8 weeks inhibited the apoptosis of retinal ganglion cells (RGCs) and improved the levels of oxidative stress markers in the retinal tissues of db/db mice (87). Since apoptosis of RGCs is the key driver for DR, astaxanthin may serve as a potential antioxidant treatment for this complication.

Astaxanthin and lutein also, respectively, showed neuroprotective effect in the retina of diabetic rats upon a supplementation of either astaxanthin (0.6 or 3 mg/kg) or lutein (0.5 mg/kg) daily for 6 weeks (Table 4). The xanthophyll carotenoids reduced ocular oxidative stress and inflammation mediated by downregulating NF-κB activity (88).

### 5.2. Clinical evidence

A few human intervention studies have been emerging recently in exploring the safety and efficacy of using carotenoids in either an oral supplement or a food form in preventing or treating diabetic complications mainly DR, DCC, and DPN (Table 5). In a case–control study involving 30 patients with non-proliferative diabetic retinopathy (NDR), a 3-month dose of mixed lutein (6 mg/d) and zeaxanthin (0.5 mg/d) supplement improved the visual acuity, contrast sensitivity (CS) and macular edema of the DR patients, concomitantly with increased serum levels of lutein and zeaxanthin, suggesting that the mixed xanthophylls possess a therapeutic potential in treating NDR. Regarding the safety of consumption, one patient reported having headache after the supplement administration, which was possibly due to allergy to the compound mixture, as explained by the researchers, but the exact reason was unknown (97). The effects of lutein and zeaxanthin together in preventing DR was also examined in a 2-year intervention study among 60 patients with T2DM. A supplement complex containing lutein (10 mg), zeaxanthin (2 mg) and meso-zeaxanthin (10 mg) resulted in a rise in the central foveal thickness and the multifocal electroretinography revealed increased retinal response density within the central 13° surrounding the fovea (rings 1–3) at 2 years of the supplementation (98). The results establish a sound foundation in building future trials to assess the long-term efficacy of using carotenoid supplementation in improving the visual function of type 2 diabetic patients. Similarly, another study using the supplement of lutein and zeaxanthin together with antioxidants (vitamin Cand mixed tocotrienols/tocopherols) and selected botanical extracts delivered in a capsule form significantly improved the visual function and the macular pigment optical density (MPOD) and ameliorated the peripheral neuropathy and the hsCRP level in patients with diabetes, both with and without retinopathy. The supplement also optimized the lipid profile of the patients by lowering LDL-C and triglycerides, with no effect on glycemic control (99). Although the study did not assess the bio-availability of individual compounds contained in the mixture upon ingestion, the increment of MPOD readings from baseline measures indicated the retinal uptake of lutein and zeaxanthin. Such multi-component formula may exert synergistic or inhibitory constituent effects, yet without a clear picture of the constituent bio-availabilities, the interpretation of the mechanistic basis is limited. The study was single-centered with a short duration, which warrants future research employing a wider patient community and longer evaluation time. In a comparable study setting, lutein was supplemented alone to 15 patients with NDR at 10 mg/day for 36 weeks. The treatment elevated the CS at four spatial frequencies, with the increment significantly exceeding that of the placebo group at 3 cycles/degree. Although not significant, a slight increase in glare sensitivity (GS) in the lutein group was also observed between baseline and week 36 (100). Lutein supplement might benefit as an adjunct therapy in preventing vision deterioration and inhibit advancement of DR in diabetic patients, yet such notion needs to be further confirmed in a larger-scaled clinical trial.

**Table 5:** Protective effects of carotenoids against diabetic complications—clinical evidence.

Carotenoid name	Administration	Experimental design	Treatment	Duration	Results		References
					Upregulation	Downregulation	
Lutein and zeaxanthin	Oral, dietary supplement	Case control in 30 patients with NDR	6 mg lutein / day 0.5 mg zeaxanthin/day	3 months	1. Increased serum levels of lutein and zeaxanthin 2. Increased visual acuity 3. Improved CS	Decreased foveal thickness	(97)
Lutein and zeaxanthin	Oral, dietary supplement	Dietary intervention in 60 patients with T2DM	lutein (10 mg), zeaxanthin (2 mg) and meso-zeaxanthin (10 mg) once a day	2 years	1. Increased central foveal thickness 2. Improved macular function assessed by mfERG		(98)
Lutein and zeaxanthin	Oral, dietary supplement	Double-blind RCT in patients with T1/2 DM with no retinopathy or mild to moderate non-proliferative retinopathy	A multi-component capsule containing lutein and zeaxanthin, antioxidants and selected botanical extracts; twice daily	6 months	Improved visual function and MPOD	1. Reduction in the serum levels of HDL-C, LDL-C and triglycerides 2. Decreased hsCRP levels 3. Reduction in DPNSSs	(99)
Lutein	Oral, dietary supplement	Double-blind RCT in 15 patients with NDR	1 capsule daily containing 10 mg of lutein	36 weeks	Increased CS at four spatial frequencies and improved CS at 3 cycles/degree		(100)
Lycopene	Oral, dietary supplement	Double blind RCT in 16 patients with T2DM	10 mg/day	2 months	1. Increased serum lycopene levels 2. Increased ratio of TAC to MDA	1. Level of serum IgG reduced when serum concentration of lycopene increased 2. Insignificant decrement in serum ox-LDL and IgG levels	(101)
Lycopene	Oral, dietary supplement	Parallel RCT in 15 patients with T2DM	250 ml tomato juice twice daily	4 weeks	1. Almost 3-fold increase in plasma lycopene levels 2. A 42% increment in the lag time in the isolated LDL oxidation by copper ions	Decreased plasma CRP levels	(102)
β-Carotene	Oral, food	Double blind, crossover RCT in 51 patients with T2DM	Fortified synbiotic food containing 0.05 g β-carotene	6 weeks	Elevated plasma nitric oxide and GSH levels	Decreased levels of insulin, HOMA-IR, HOMA-B, triglycerides and VLDL-C and TC/HDL-C ratio	(103)
β-Carotene, α-tocopherol	Oral, dietary supplement	Double-blind 2×2 factorial design RCT in 29,133 male-smokers (1,700 with T2DM—662 diagnosed with first-ever macrovascular complication)	1 capsule daily, either containing α-tocopherol (50 mg/d), β-carotene (20 mg/d), or both	3 years	No effect	No effect	(104)
Carotenoids	Oral, dietary supplement	Parallel RCT in 21 patients with T2DM	40 g green banana biomass added to habitual diet daily	6 months	Increased carotenoid content in the LDL-C particles, i.e., enhanced prevention of LDL-C oxidation	Reduced levels of TC, non-HDL-C, glucose and HbA1c	(38)

NDR, nonproliferative diabetic retinopathy; CS, contrast sensitivity; T2DM, type-2 diabetes mellitus; mfERG, multifocal electroretinography; RCT, randomized controlled trial; MPOD, macular pigment optical density; HDL-C, high-density lipoprotein cholesterol; LDL-C, low-density lipoprotein cholesterol; hsCRP, high-sensitivity C-reactive protein; DPNSSs, diabetic peripheral neuropathy symptom scores; TAC, total antioxidant capacity; MDA, malondialdehyde; IgG, immunoglobulin G; ox-LDL, oxidized-LDL; GSH, glutathione; HOMA-IR, homeostatic model assessment of insulin resistance; HOMA-B, homeostatic model assessment of β-cell function; VDL-C, very-low-density-lipoprotein-cholesterol; TC, total cholesterol; HDL-C, high-density lipoprotein cholesterol; HbA1C, glycosylated hemoglobin.

Echoing the animal study findings, lycopene administered in an oral supplement or whole-food form showed cardioprotective effect among patients with T2DM (Table 5). A 10 mg/day supplementation for 2 months attenuated T cell-dependent adaptive (pro-atherogenic) immune response, and prevented the oxidized-LDL uptake by macrophage and foam cell formation, accompanied with increased serum lycopene levels and antioxidant capacity among 16 patients with T2DM (101). Comparatively, when lycopene taken as 250 ml tomato juice twice daily among 15 patients with T2DM, the intrinsic resistance of LDL to oxidation increased almost as effectively as supplementation with a high dose of vitamin E and the plasma levels of CRP in patients also declined, together with an elevation in plasma lycopene levels (102). The results are suggestive on the preventive role of lycopene against long-term diabetic complications, notably CVD. β-Carotene ameliorated glucose intolerance and improved the plasma lipid profile and NO and GSH levels in 51 patients with T2DM when consumed in a fortified synbiotic food containing 0.05 g β-carotene (103). Although the results favor a conclusion on β-carotene’s antidiabetic role, its cardioprotective effect was not confirmed in a large randomized controlled trial (RCT) (104). As part of the α-Tocopherol, β-Carotene Cancer Prevention Study, 29,133 middle-aged male smokers received either vitamin E (50 mg/day) or β-carotene (20 mg/day), or both, or placebo for a median of 6.1 years. The cohort (1700 men) had ~5% being diagnosed withtT2DM, among whom 662 had the first-onset macrovascular complications. Neither intervention made any changes in the risk of macrovascular complication or total mortality. It was concluded that β-carotene or α-tocopherol supplementation has no protective effect on macrovascular outcomes or total mortality of diabetic male smokers (104). However, the study result cannot be generalized to women and non-smokers, and the specificity of its design toward diabetic complication prevention is debatable, as the study was primarily targeted at examining the effects of those antioxidants toward lung cancer (104), hence influencing the validity/suitability of the supplement’s dosage, timing and duration in treating diabetes. Contrarily, carotenoids, when taken in a whole food, did present protective effects against the risk of developing CVD among patients with T2DM. Beta-and alpha-carotenes are the predominant forms for carotenoids found in banana fruit (105). In an intervention study, 21 patients with T2DM took 40 g of green (unripe) banana biomass daily for 6 months experienced considerable changes in CVD risk factors, including improved blood lipid profile and glucose tolerance level as well as enhanced protection of the LDL particles against oxidation mediated by an increased carotenoids content in those particles (105). The effective protection against LDL-modifications implies a decreased risk of developing CVD. The glycemic control and anti-dyslipidemia functions of unripe banana were also likely to be partly attributed to its resistance starch content, which has exhibited similar antidiabetic effects upon consumption in both prediabetic and diabetic patients (106). In addition, no adverse events such as diarrhea, constipation and abdominal bloating were reported throughout the intervention, indicating the high tolerability of green banana biomass (38). The efficacy and safety of the green-banana biomass encourage its use as potential therapeutics for patients with T2DM.

## 6. Discrepancy between outcomes of animal experiments and human trials

Recent meta-analyses of prospective cohort studies have confirmed that intake of flavonoid-rich foods is associated with a lower risk of T2DM. In the studies, intake of flavonoids was calculated using food frequency questionnaires and data from the United States Department of Agriculture^1^ or from the European Phenol-Explorer Database.^2^ A significant correlation was found between the intake of anthocyanidins, flavan-3-ols, flavonols, and isoflavones, however, no significant association was observed with respect to either flavanones or flavones (29, 107–109).

Though the cohort studies indicate a beneficial effect of flavonoids, the mechanism of action has remained elusive. Initial studies pointed at the antioxidant activities of flavonoids as a potential mechanism to prevent diabetic complications caused by chronic hyperglycemia. However, the poor bioavailability of flavonoids rarely makes the levels reached in the blood serum or in systemic circulation that are sufficient to give an *in vivo* antioxidant effect. Admittedly, flavonoids may trigger antioxidant signaling pathways and thus stimulate production of endogenous antioxidants.

An alternative mechanism that may explain the preventive effect of flavonoids against the onset of diabetes may be that they act as α-glucosidase inhibitors in the digestive tract, much like the prescription drug acarbose. Thus, dietary flavonoids may inhibit the absorption of carbohydrates from the small intestine, resulting in lowered blood sugar levels (110, 111). Flavonoids in the digestive tract affect, or alter the composition of the resident gut microflora. The microbiota-gut-brain axis is drawing increasing attention as an important system that connects nerve, hormone and immune signals, and thus regulates human metabolism (28, 111). At present, the role of gut microflora in prevention of diabetes is still a matter of speculation.

Meta-analysis of cross-sectional studies and randomized controlled trials linking serum levels of total carotenoids and vitamin A with metabolic syndrome, showed an inverse association. This association was strongest for β-carotene, followed by α-carotene and β-crypotoxanthin. No significant association was detected between retinol and metabolic syndrome (112).

If medicinal claims are made for dietary phytochemicals, e.g., a role of flavonoids or carotenoids in the prevention or management of diabetes, then the weight of evidence that is needed to claim efficacy ought to be similar to that required in classical pharmacology. However, in nutrition studies it is generally common to ascribe biological activity to complex mixtures or to synergism. That approach would be undesirable in drug design and classical pharmacology and is also not conducive to the elucidation of an unambiguous mechanism of action. Whereas some *in vivo* animal studies have focused on single flavonoids or carotenoids, very few human intervention studies looked at the effect of single compounds (human cohort studies do not consider single compounds at all). This may explain why proposed mechanisms of action developed from animal studies have never been unambiguously confirmed in human clinical trials.

## 7. Conclusion and future perspectives

**Figure 19:**
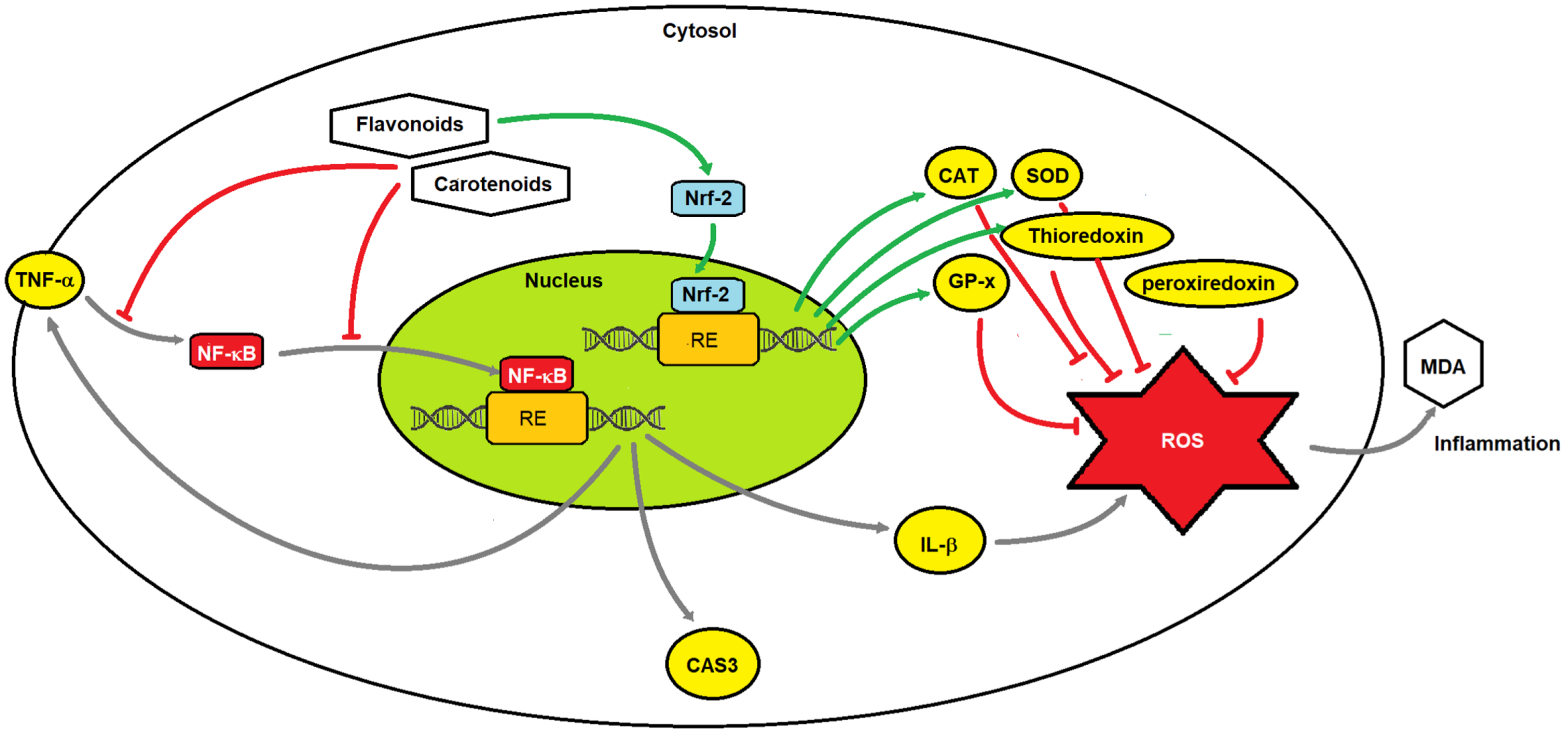
Mechanisms of the anti-inflammatory effect of flavonoids and carotenoids.

The current preclinical and clinical evidence largely supports the therapeutic roles of the two natural compound groups (flavonoids and carotenoids) in preventing the advancement of diabetic conditions and the onset and progression of diabetic complications, mainly DCC, DN, and DR. Some evidence showed carotenoids’ preventive effects against DPN, but not for flavonoids in this review. Notably, carotenoids’ protective effects are more oriented toward DR. Their modes of therapeutic actions are pleiotropic involving alleviating hyperglycemia, insulin resistance and dyslipidemia, attenuating oxidative stress and metabolic inflammation, elevating insulin sensitivity and antioxidant capacity in tissues and organs, as well as combating key risk factors of complications such as hypertension (for DCC) and increased apoptosis of RGCs (for DR). Among all, the anti-inflammatory and antioxidant properties of flavonoids and carotenoids form their principal mechanistic basis in treating diabetes and preventing/ameliorating its complications (Figure 19), given the pathogenic mechanisms driving chronic diabetic complications primarily involve metabolic inflammation and oxidative stress (113).

From the *in vivo* studies reviewed, there is a clear discrepancy in the level of dosage and length of feeding duration applied in all experimental settings including human interventions. Also, some beneficial effects may have been derived from synergistic actions of multiple phytochemical compounds administered instead of single ones. Most of the clinical trials to date studied the effect of concentrated phytochemical extracts taken as a supplement rather than that of whole foods rich in those phytochemicals. Very few investigated the bioavailability of the test compounds upon ingestion under diabetic conditions. Hence, the interactions of diabetes (specially hyperglycemia) and its complications with the bioavailability and bioactivity of those phytochemicals are not clearly understood, which limits the extension of knowledge of the mechanisms underlying their protective effects. The existence of other dietary components such as starch, fiber and alkaloids may enhance the antidiabetic potential of those phytochemicals, e.g., flavonoids (114), yet, current clinical trials tend not to consider this aspect in the study design. Also, the present clinical evidence focuses on the short-term effect rather than long term, the latter of which more fits into the development pattern of chronic diabetic complications. Therefore, appropriate and well-designed clinical trials are necessary to identify pharmacologically active dietary phytochemicals and elucidate their mechanism of action in treating diabetes-related complications when dosed as whole foods and on a long-term basis.

In summary, these naturally occurring phytochemicals hold great potential for further development into novel drugs/therapies for treating diabetes and its complications. Yet, future long-term RCTs with bioavailability investigations are needed to identify their effective dose and duration, and safety and tolerability at various doses consumed among patients at different disease stages before confident clinical applications.

## Author contributions

All authors listed have made a substantial, direct, and intellectual contribution to the work and approved it for publication.

## Conflict of interest

The authors declare that the research was conducted in the absence of any commercial or financial relationships that could be construed as a potential conflict of interest.

## Publisher’s note

All claims expressed in this article are solely those of the authors and do not necessarily represent those of their affiliated organizations, or those of the publisher, the editors and the reviewers. Any product that may be evaluated in this article, or claim that may be made by its manufacturer, is not guaranteed or endorsed by the publisher.

## Footnotes

^1^ http://www.ars.usda.gov/nutrientdata

^2^ http://phenol-explorer.eu/
